# Artificial Intelligence in Cardiovascular Medicine: A Giant Step in Personalized Medicine?

**DOI:** 10.3390/jpm16040192

**Published:** 2026-04-01

**Authors:** Stanislovas S. Jankauskas, Fahimeh Varzideh, Urna Kansakar, Gaetano Santulli

**Affiliations:** School of Medicine, City University of New York, Manhattan, NY 10031, USA

**Keywords:** artificial intelligence, cardiovascular disease, digital health, machine learning, precision medicine, predictive analytics, remote monitoring, wearable devices

## Abstract

Artificial intelligence (AI) is rapidly reshaping cardiovascular (CV) medicine, driving a paradigm shift toward truly personalized and data-driven care. This comprehensive review examines the conceptual foundations, clinical applications, and future implications of AI across the CV continuum, spanning prevention, diagnosis, risk stratification, and therapy. Core AI methodologies (including machine learning, deep learning, natural language processing, and computer vision) are discussed in the context of cardiology’s uniquely data-rich environment, encompassing imaging, electrocardiography, electronic health records, wearable devices, and multi-omics data. This systematic review highlights major clinical domains where AI has demonstrated a substantial impact, including CV imaging, ECG interpretation, hypertension and heart failure management, coronary artery disease, acute coronary syndromes, interventional cardiology, and cardiac surgery. AI-driven predictive analytics enable early detection of subclinical disease, improved prognostication, and individualized prevention strategies, while wearable technologies and remote monitoring platforms facilitate continuous, real-world patient surveillance. Emerging applications in pharmacotherapy, drug repurposing, and genomics further reinforce AI’s role in advancing precision cardiology. Equally emphasized are the ethical, legal, and social challenges accompanying AI adoption, such as algorithmic bias, data privacy, cybersecurity, interpretability, and regulatory oversight. Our review underscores the necessity of rigorous clinical validation, transparent model design, and seamless integration into clinical workflows to ensure safety, equity, and physician trust. Ultimately, AI is best positioned as an augmentative tool that complements (but does not replace!) clinical expertise. By fostering hybrid intelligence that integrates human judgment with computational power, AI has the potential to redefine CV care delivery, improve outcomes, and support a more proactive, patient-centered healthcare model.

## 1. Introduction

The rise of Artificial Intelligence (AI) in cardiovascular (CV) medicine marks a significant evolution in healthcare, aligning closely with historical advancements in AI across the broader medical landscape. To understand how AI is transforming CV medicine, it is essential to explore the historical context of AI in healthcare (Table 1), the unique attributes that make cardiology conducive to AI adoption, and the current momentum driving this transformation.

AI’s journey in healthcare began with the development of expert systems in the 1970s and 1980s, which were designed to assist with diagnosis through a rule-based approach. These early systems, although limited by the technology of their time, laid the foundation for subsequent advancements in machine learning (ML) and natural language processing (NLP) applications in the healthcare sector [51,52]. As computational capabilities improved, AI began to leverage vast datasets, including electronic health records and imaging data. Milestones included the emergence of diagnostic algorithms that utilized deep learning (DL) techniques to enhance image interpretation and patient management strategies [53,54]. The evolution of AI in healthcare has coincided with a growing acknowledgment of the need for evidence-based medicine, where AI tools are increasingly integrated into workflows to facilitate more accurate clinical decision-making and enhance patient outcomes [55].

Cardiology, with its complex data types (including imaging, electrophysiology, and genomic information) presents a unique landscape well-suited for AI adoption. The volume and variability of CV data require efficient analytical methods capable of extracting meaningful patterns from large, heterogeneous datasets. Modern CV research and clinical practice generate vast amounts of multimodal information, including imaging data, genomic and transcriptomic profiles, biomarker measurements, wearable device outputs, and longitudinal clinical records. These data sources differ in structure, scale, and temporal resolution, creating substantial analytical complexity. Advanced computational approaches, including machine learning and integrative data modeling, enable the identification of subtle associations, predictive signatures, and mechanistic pathways that may not be apparent using conventional statistical techniques. By facilitating the integration and interpretation of complex CV datasets, these analytical frameworks can support earlier diagnosis, improved risk stratification, and the development of more targeted therapeutic strategies [56,57]. In cardiology, AI applications range from risk stratification models predicting patient outcomes to automated interpretation of echocardiograms and cardiac MRIs, thus significantly improving diagnostic accuracy and facilitating personalized treatment [58]. This integration supports cardiologists in improving clinical efficiency and enhancing the quality of care delivered to patients [59]. By automating routine tasks, AI frees up valuable time for clinicians, enabling them to focus on direct patient interactions and complex decision-making.

The current drive behind AI in CV medicine is mainly characterized by transformative trends that enhance both clinical practice and research. The proliferation of AI-driven diagnostic tools has led to a clearer understanding of cardiac pathologies by aiding in early detection and intervention strategies [60,61]. Continuous monitoring technologies, often supported by wearable devices, utilize AI to collect real-time data on patients, allowing for proactive management of conditions such as hypertension and heart failure (HF) [57]. AI’s role in clinical trials has been pivotal in accelerating drug discovery and therapeutic innovation, indicating a trajectory toward a more iterative and data-driven approach to treatment advancements [62]. This convergence of digital technologies underscores a broader shift toward a patient-centric model of care that aligns with contemporary health priorities and fosters greater engagement between patients and healthcare providers [63].

The transformative potential of AI in cardiology extends beyond diagnostic and treatment enhancements; it also encompasses operational improvements within healthcare systems. AI algorithms can improve healthcare delivery systems by predicting patient admission patterns and optimizing resource management to reduce waste and inefficiencies [64,65]. These innovations are crucial given the increasing demands on healthcare systems worldwide due to the aging population and rising prevalence of CV diseases [56,66]. Stakeholders in healthcare should navigate this new terrain with careful attention to ethical considerations, ensuring that AI technologies are deployed responsibly, transparently, and equitably in all areas of practice [67,68].

In the context of equity in healthcare, AI must also address fundamental issues such as algorithmic bias, data privacy, and the need for inclusive datasets that reflect diverse populations [69,70]. These challenges highlight the dual-edged nature of AI’s potential and necessitate policies that foster a holistic understanding of how reliable, fair, and inclusive AI should be within the landscape of CV care. So, the rise of AI in CV medicine illustrates a profound shift underpinned by historical advancements in AI technology, the inherent qualities of cardiology as a specialty for AI applications, and the transformative momentum currently reshaping the field. As healthcare providers and organizations continue to harness AI’s capabilities, it is crucial to remain vigilant in addressing ethical concerns and ensuring that these technological innovations prioritize patient welfare and equitable access to care. The prospective horizons of AI in CV medicine promise not only to enhance diagnostic and therapeutic capabilities but also to revolutionize how patient care is conceptualized, delivered, and experienced across the healthcare continuum.

Several ethical concerns arise with the implementation of AI in CV medicine and healthcare systems. One major issue is algorithmic bias, which can occur when AI models are trained on datasets that do not adequately represent diverse patient populations, potentially leading to unequal diagnostic accuracy or treatment recommendations across demographic groups. Data privacy and security also represent critical challenges, as AI systems rely on large volumes of sensitive clinical information, including imaging, electronic health records, and molecular data, which must be protected from misuse or unauthorized access. Transparency and explainability are additional concerns, since many AI models operate as complex “black boxes,” making it difficult for clinicians to understand the reasoning behind specific predictions or recommendations. Questions of accountability and liability may arise when AI-assisted decisions contribute to clinical errors, creating uncertainty regarding responsibility among clinicians, institutions, and developers. Furthermore, careful attention must be paid to informed consent for data use and to ensuring equitable access to AI technologies so that these innovations do not exacerbate existing disparities in healthcare delivery.

## 2. Foundations of AI

The foundations of AI, particularly in the realm of ML and DL, are pivotal in enhancing medical practices, especially in cardiology (Table 2).

Rule-based systems are knowledge-based AI tools that apply predefined “if–then” logical rules derived from expert consensus or clinical guidelines to assist with diagnosis and management. They have been used to detect myocardial infarction from ECG patterns, support anticoagulation decisions in atrial fibrillation, and prompt evidence-based therapies such as β-blocker initiation after myocardial infarction. These systems are transparent and easily interpretable, offering reliable adherence to established protocols, but they lack flexibility and adaptability when faced with complex or atypical clinical presentations, and require continuous manual updating as CV knowledge evolves.

Supervised learning is a type of ML where the algorithm is trained on labeled datasets, containing input-output pairs, which allows the model to learn a mapping from inputs to the correct outputs [86]. This approach is particularly effective in diagnostic applications where labeled data, such as medical images with corresponding disease states, can teach the models the key characteristics of various conditions. For instance, Convolutional Neural Networks (CNNs), a classic example of supervised learning, have been employed in analyzing cardiac MRI scans to identify abnormalities that denote potential CV diseases [87,88,89]. Unsupervised learning, in contrast, deals with unlabeled data and aims to identify hidden patterns without explicit instructions on what to find [86]. This technique can be particularly valuable in cardiology for clustering patients based on similarities in physiological data, aiding in risk stratification and personalized treatment [86].

Reinforcement learning (RL) presents a third paradigm where an algorithm learns to make decisions by performing actions within an environment to maximize cumulative rewards [90]. In the context of cardiology, RL can optimize treatment pathways by adjusting interventions based on patient responses, thus personalizing care plans for conditions such as HF or diabetes-related CV complications [88]. The application of RL in mobile health technology showcases its potential in promoting adherence to treatment protocols by using feedback mechanisms that guide patient interactions based on their adherence patterns [88].

NLP enables computers to interpret and extract clinically meaningful information from unstructured text such as electronic health records, imaging reports, and clinical notes. By applying linguistic and ML techniques, NLP can identify CV diagnoses, risk factors, medication use, and procedural outcomes that are often not captured in structured fields. It has been used to automatically detect HF phenotypes, extract echocardiographic parameters, and summarize cardiology consults. NLP enhances data retrieval, supports clinical research, and facilitates automated documentation, but its performance can be limited by variability in clinical language, data quality, and the need for domain-specific adaptation to maintain accuracy and reduce misinterpretation.

Computer vision (CV) involves the use of DL and image analysis algorithms to interpret visual data such as echocardiograms, cardiac CT, MRI, and angiographic images. By recognizing spatial patterns and subtle features beyond human perception, CV systems can automate tasks such as left ventricular function quantification, plaque characterization, and detection of structural abnormalities. They have demonstrated high accuracy in identifying coronary artery disease (CAD), classifying cardiomyopathies, and assessing valvular lesions. CV improves efficiency, consistency, and diagnostic precision, yet its performance can be affected by image quality, variations in acquisition protocols, and limited generalizability across populations or devices, necessitating careful validation and clinician oversight.

The architectures of DL play a significant role in medical AI applications. CNNs have proven vital in processing grid-like topology data, such as images, where they excel in detecting spatial hierarchies of features, thus leading to advancements in image recognition tasks like detecting arrhythmias from ECG signals [87]. Recurrent Neural Networks (RNNs) are designed to handle sequential data, making them ideal for time-series analysis, which is essential in cardiology when analyzing patients’ historical health records to detect temporal patterns in cardiac function [91]. More recently, transformers, which leverage attention mechanisms to model relationships between different data points, have been gaining traction. Their capabilities in natural language processing are translating to significant advances in the automated summarization of medical chart notes, enabling more efficient data extraction and insight generation in cardiology practices [92]. However, a significant challenge arises concerning interpretability. The “black box” nature of many DL models, where the decision-making process is not transparent to users, raises concerns, particularly in clinical settings where understanding the rationale behind predictions is essential for patient safety and trust. Explainable AI (XAI) seeks to address this challenge by providing frameworks that make the outputs of AI systems interpretable. For instance, methods such as SHAP (SHapley Additive exPlanations) allow clinicians to understand how specific input features contribute to the model’s predictions, indicating which anatomical features in a cardiac MRI are driving a decision towards diagnosing heart disease [93].

In the cardiology landscape, the balance between leveraging powerful DL models and ensuring their interpretability reflects a broader ethical commitment to patient-centered care. AI applications must not only focus on enhancing diagnostic accuracy but also facilitate informed clinical decision-making through transparent and understandable AI interventions. This dual focus is critical as it fosters trust among healthcare providers and patients, thus enabling the broader adoption of AI technologies in clinical cardiology.

With the integration of federated learning, a decentralized approach that utilizes local data to train models without transferring sensitive information to a centralized server, there is a growing opportunity to enhance model performance while maintaining patient confidentiality and compliance with healthcare regulations [12,47,48,94,95,96,97]. This approach is instrumental in addressing ethical dilemmas and privacy concerns associated with AI in healthcare, particularly when dealing with sensitive patient data.

The foundations of AI have profound implications for the field of cardiology. The transition from traditional diagnostic methods to AI-enabled solutions signifies a substantial leap forward, characterized by enhanced accuracy, efficiency, and personalization of patient care. However, the imperative to ensure model transparency highlights the need for a balanced approach to adopting these technologies, with the ultimate goal of harmonizing innovation and the ethical dimensions of clinical practice. Through continued advancements in AI technology accompanied by robust frameworks for interpretation and patient engagement, the future of cardiology stands to become increasingly data-driven while remaining compassionate and patient-centric.

## 3. Big Data in Cardiology: Unlocking Clinical Insights

Big Data has become a transformative force in cardiology, fostering the potential to unlock clinical insights that can significantly enhance the prevention, diagnosis, and treatment of CV diseases. The sources of CV data are diverse, encompassing Electronic Health Records (EHRs), patient registries, medical imaging, and wearable devices. Each of these sources contributes unique information that, when synthesized through big data analytics, provides a more comprehensive understanding of CV health.

EHRs have emerged as a cornerstone of big data in healthcare, containing extensive patient information, including demographics, medical histories, laboratory results, and treatment outcomes [98]. These records facilitate longitudinal studies that can identify trends over time and assist in risk assessments for CV diseases. Registries, often specifically tailored to certain conditions such as HF or myocardial infarction, aggregate data from multiple institutions and provide invaluable insights into treatment patterns, patient responses, and long-term outcomes [99]. Medical imaging technologies further enrich CV datasets, producing vast amounts of visual data from echocardiograms, MRIs, and CT scans, enhancing diagnostic capabilities and treatment planning [100].

Wearable devices have gained rapid integration into cardiology, enabling continuous monitoring of patients’ vital signs, physical activity, and other health markers [101,102,103,104,105,106,107,108,109,110,111,112,113,114,115,116,117,118,119,120,121,122,123,124,125]. This real-time data collection allows for proactive management of CV health, enabling clinicians to identify deteriorations in health before they manifest as acute events. The integration of data sources creates a wealth of information that can be harnessed through sophisticated analytical techniques to derive actionable insights, enhancing the overall quality of care.

However, the journey from data accumulation to meaningful insights is fraught with challenges, particularly in data preprocessing, integration, and interoperability. Preprocessing is crucial, as it involves cleaning, normalizing, and transforming data to ensure it is suitable for analysis [126]. Given the vast volume and variety of data sources, achieving consistency in data formats and content is a daunting task. Integration involves consolidating disparate data streams into a unified system, which can be hindered by varying standards and regulatory requirements across different healthcare systems [127].

Interoperability remains a critical barrier; different healthcare organizations often use incompatible systems that inhibit the seamless exchange of information [128]. This fragmentation complicates the ability of healthcare providers to gain a holistic view of patient health across multiple data sources. Solutions like standardized data formats and application programming interfaces (APIs) are essential in fostering interoperability, enabling disparate systems to communicate effectively [129]. Legislative support for initiatives promoting health information exchange can also facilitate better data sharing practices, leading to improved patient outcomes.

Cloud computing has emerged as a vital enabler for big data analytics in cardiology, providing the necessary infrastructure for storing and processing vast amounts of data. The scalability and flexibility of cloud services allow healthcare organizations to manage their data needs without significant capital investment in physical infrastructure [130]. Cloud computing solutions support advanced analytics that can drive predictive modeling and ML efforts, allowing clinicians to proactively manage patient care based on data-driven insights [98]. Federated learning is a promising approach that addresses both data privacy and interoperability concerns. By allowing algorithms to learn from decentralized data sources without requiring the data to leave its original location, federated learning promotes collaboration across healthcare institutions while preserving patient confidentiality [131]. This approach can improve the robustness of predictive models, as the algorithms are trained on a more diverse set of data, leading to better generalizability across patient populations [132]. As federated learning continues to evolve, it will likely play a significant role in future cardiology applications, enabling data-driven innovations while respecting patient privacy.

## 4. AI in CV Imaging

AI is rapidly transforming CV imaging, enhancing accuracy, speed, and reproducibility in diagnosis and management (Table 3). In particular, AI applications in echocardiography, cardiac computed tomography (CT), cardiac magnetic resonance imaging (MRI), and nuclear imaging are revolutionary, allowing for automated measurements, disease detection, and significant clinical insights.

### 4.1. Echocardiography: Automated Measurements and Disease Detection

Echocardiography is a critical modality in cardiac imaging, providing real-time assessment of cardiac structure and function without the need for ionizing radiation. Recent developments in AI have catalyzed the automation of measurements in echocardiography, facilitating rapid evaluations of left ventricular ejection fraction (LVEF) and other cardiac parameters [170,171]. The introduction of automated three-dimensional echocardiography has improved consistency and reproducibility of left ventricular volume assessments compared to traditional two-dimensional techniques [172]. Automated algorithms enable high-throughput analysis, providing accurate measurements of important indices such as mitral valve area or left atrial size, which are crucial in managing various cardiac conditions [173,174].

AI has also shown great potential in disease detection. It has been implemented for assessing conditions like pulmonary hypertension by evaluating the tricuspid regurgitation jet velocity with high accuracy [175]. Systems are now capable of detecting subtle changes in cardiac morphology that might indicate pathologies, facilitating earlier diagnosis and treatment initiation. AI-assisted echocardiographic systems can enhance patient stratification, guiding clinical decision-making by flagging patients at risk for conditions such as HF or valvular heart diseases [176].

An example of advances in this field is given by studies evaluating the efficacy of automated echocardiographic assessments in pediatric patients, showcasing the methodology’s advantages in complex scenarios like those involving extracorporeal membrane oxygenation [177]. Overall, automation not only improves efficiency but also empowers non-specialists to perform echocardiographic assessments effectively, extending the use of echocardiography in diverse healthcare settings [178].

### 4.2. Cardiac CT: Plaque Characterization and Coronary Anatomy

Moving to cardiac CT, AI is significantly enhancing the analysis and interpretation of coronary anatomy and plaque characterization. CT imaging, particularly coronary computed tomography angiography (CTCA), is instrumental in non-invasively visualizing coronary arteries, providing insights into CAD. ML algorithms have been developed to differentiate between various types of plaques (calcified, non-calcified, and mixed plaques) allowing for precise risk stratification and treatment planning [179,180]. Automation in image interpretation helps reduce inter-reader variability and enhances diagnostic consistency [181].

DL models are useful in improving the accuracy of detecting coronary artery stenoses, which is critical for timely interventions [182]. AI algorithms can analyze complex imaging data swiftly, identifying structures and conditions that might be missed by human readers. This capability speeds up the diagnostic process and contributes to enhanced clinical confidence in imaging findings, ultimately improving patient care and outcomes [183,184].

### 4.3. Cardiac MRI: Tissue Characterization and Functional Analysis

Cardiac MRI is revered for its superior tissue characterization and functional analysis capabilities. It provides unparalleled insights into myocardial perfusion, edema, and fibrosis, critical in the assessment of various cardiomyopathies and ischemic heart disease. The integration of AI in this domain aids in automating the segmentation of the left ventricle, facilitating precise function quantification without the variability associated with manual image analysis [185,186]. Recent advancements have also included coupling AI with traditional imaging techniques, enabling improved myocardial strain assessment, a key indicator of cardiac function [187,188].

AI also assists in the interpretation of late gadolinium enhancement patterns, crucial for diagnosing myocardial infarction and differentiating between ischemic and non-ischemic cardiomyopathy [189,190]. AI-driven analysis allows clinicians to classify images more efficiently, thus yielding faster results and reducing the time patients wait for critical diagnoses. However, while AI automates traditionally labor-intensive tasks, it is paramount that healthcare professionals remain engaged in the interpretive process to integrate clinical correlation and judgment [191,192].

### 4.4. Nuclear Imaging: Perfusion Quantification and Hybrid Imaging

Nuclear imaging modalities, such as positron emission tomography (PET) and single-photon emission computed tomography (SPECT), offer essential insights into myocardial perfusion and viability. AI applications in this field focus on optimizing the quantification of myocardial perfusion defects, improving sensitivity and specificity in detecting CAD [193,194]. AI algorithms can analyze SPECT images to quantify regional myocardial blood flow changes effectively, yielding volumetric assessments that guide clinical decisions regarding revascularization or medical therapy [180,190].

Hybrid imaging approaches, combining CT or MRI with nuclear modalities, benefit significantly from AI. Integration of dual-modality imaging, particularly CT with SPECT or PET, creates more comprehensive datasets, leading to better diagnostic accuracy [195,196]. AI enhances the interpretation of these complex datasets by providing advanced visualization tools that allow for more nuanced analysis of underlying cardiac pathology [179,197]. As the landscape of cardiac diagnostics evolves, the fusion of imaging modalities with AI will likely result in more effective patient-centered solutions and treatment pathways.

Hence, the integration of AI into CV imaging is transforming the diagnostic landscape across various imaging modalities including echocardiography, cardiac CT, cardiac MRI, and nuclear imaging. Each modality embraces AI’s capabilities for automating measurements, enhancing disease detection, and improving tissue characterization and functional analysis. As technologies continue to evolve, it is imperative to balance automation with human expertise to ensure comprehensive patient care. Future advancements will likely further establish AI as an indispensable asset in optimizing CV imaging practices, improving clinical outcomes and patient safety.

## 5. Electrocardiogram (ECG) Analysis with AI

AI is making significant strides in the field of cardiology, particularly through enhanced ECG analysis. This progression is evident in the applications of AI for arrhythmia detection, uncovering hidden phenotypes related to left ventricular dysfunction, and ischemia prediction, as well as the implementation of continuous monitoring via wearable ECG devices.

### 5.1. Arrhythmia Detection: Atrial Fibrillation and Ventricular Tachyarrhythmias

One of the primary successes of AI in ECG analysis is its capability to detect arrhythmias, particularly atrial fibrillation (AF) and ventricular tachyarrhythmias. Traditional ECG interpretation often faces challenges due to variability in patient presentations, and AI algorithms have shown promise in improving the sensitivity and specificity of arrhythmia detection [198,199]. DL frameworks, such as CNNs, can analyze ECG signals to accurately identify AF, even when the arrhythmia is intermittent. For instance, models based on CNNs have been reported to achieve an accuracy of 95.3% in detecting AF when applied to Holter monitoring data, illustrating their potential for reliable clinical decision support [200,201].

AI advancements also extend to identifying other life-threatening arrhythmias beyond AF, such as ventricular tachycardia. AI’s ability to recognize complex patterns in ECG readings allows for real-time monitoring and immediate alerting of healthcare providers when abnormal rhythms are detected, thus enhancing patient safety and response measures during emergencies [202]. Integrating AI-enhanced algorithms can significantly reduce false-positive rates for arrhythmia detection when used in conjunction with traditional monitoring systems [199].

AI contributes significantly to the continuity of care by providing ongoing assessments of cardiac health. This is particularly relevant in post-procedural monitoring scenarios where patients may be at heightened risk for arrhythmias [202,203]. The potential for automated detection and alerting can relieve the workload on medical staff, enabling them to focus on higher-priority patient care activities.

### 5.2. Hidden Phenotypes: Left Ventricular Dysfunction and Ischemia Prediction

The role of AI in ECG analysis also encompasses detecting hidden phenotypes that signify underlying cardiac conditions. Left ventricular (LV) dysfunction and ischemic conditions can manifest through subtle changes in the ECG that may be overlooked during standard evaluations. AI algorithms can analyze these nuanced variations to predict ischemia and assess LV dynamics [198,204]. AI models can extract features from ECG data that correlate with decreased ejection fraction (EF), enhancing the ability to risk-stratify patients early [205].

AI techniques can utilize longitudinal ECG data to monitor patients over time, allowing for the identification of trends related to LV dysfunction that may not be apparent in isolated measurements. This capacity is critical for guiding early interventions and adjusting treatment strategies in individuals with chronic heart conditions [206]. Such predictive capabilities signify a shift from merely reactive cardiology to a more proactive and preventive approach based on data-driven insights.

### 5.3. Continuous Monitoring with Patch-Based or Wearable ECG Devices

The integration of AI with continuous monitoring technologies is revolutionizing CV health management. Wearable ECG devices, such as patch-based systems, enable real-time monitoring of patients’ heart rhythms, facilitating the early detection of arrhythmias and other CV events in non-clinical settings [203,207]. These devices, powered by AI algorithms, yield diagnostic accuracy that matches or exceeds traditional methodologies, providing greater accessibility to vital health insights for patients in their everyday environments [208,209].

Research involving smartphone-integrated ECG devices illustrates how AI can facilitate continuous, remote monitoring, promoting not only the early detection of conditions like AF but also enabling chronic condition management [203,210]. The adoption of such technologies has been particularly beneficial for managing patient populations with a high burden of symptomatic heart disease, improving adherence to treatment plans and enhancing patient engagement in their care [198,211].

The utility of AI in wearable technology extends beyond mere arrhythmia detection; it includes comprehensive data analytics that can inform lifestyle modifications and preventive health measures. By analyzing data trends, AI-driven platforms can generate personalized health recommendations and alert patients or healthcare providers about significant changes in their heart rhythms, which is crucial during recovery phases following cardiac events [207,212].

Thus, the application of AI in ECG analysis holds promise for improving CV care across multiple dimensions, particularly in arrhythmia detection, assessment of hidden cardiac phenotypes, and continuous monitoring via wearable devices. As AI methods mature, the potential for enhancing diagnostic accuracy and clinical decision-making will continue to grow, which may lead to significant impacts on patient management and outcomes in cardiology. Future developments should focus on refining algorithmic approaches, improving integration with healthcare systems, and addressing the regulatory and ethical considerations inherent in deploying AI technologies in cardiology.

## 6. Predictive Analytics for CV Risk

Predictive analytics for CV risk is a rapidly evolving field that employs sophisticated algorithms and data-driven methodologies to improve the identification, stratification, and management of CV diseases. These advancements stand in contrast to traditional risk calculators, offering more nuanced insights and the potential for personalized patient care.

### 6.1. Risk Calculators vs. AI-Driven Models

Traditional CV risk calculators, such as the Framingham Risk Score, have been foundational in assessing the likelihood of events like heart attacks and strokes based on established risk factors including age, cholesterol levels, blood pressure (BP), smoking status, and diabetes [213]. These calculators are widely used in clinical practice due to their simplicity and the ability to apply practical guidelines across diverse populations [214]. However, they have limitations concerning accuracy, often failing to incorporate a multitude of emerging risk factors and patient-specific variables that can significantly influence risk [213,215]. In contrast, AI-driven models leverage advanced ML and DL techniques to refine CV risk predictions. These models can analyze vast datasets, including clinical data, genetic information, imaging studies, and even social determinants of health, providing a multifaceted view that can lead to more accurate predictions [216]. For example, the incorporation of advanced imaging data such as coronary artery calcium scores into AI models has demonstrated enhanced predictive capabilities compared to traditional scores [217]. ML techniques have shown improvements in predictive accuracy, with studies reporting enhancements in risk prediction by up to 3.6% when comparing conventional methods to AI algorithms [218]. AI models also have the capacity to learn from incoming data dynamically, adapting in real-time and improving prediction quality as more data becomes available. For instance, researchers have shown that using AI can yield specific insights about subclinical disease and risk patterns in populations that traditional models might not detect, allowing clinicians to take preemptive action [216]. Thus, while risk calculators are useful for initial assessments, AI-driven models facilitate a more comprehensive and personalized approach to CV risk assessment.

### 6.2. Early Detection of Subclinical Disease

One of the most significant advances in predictive analytics is the ability to identify subclinical CV disease, which often precedes clinical symptoms and events. AI algorithms can analyze patterns in ECGs or imaging studies to detect changes that may indicate left ventricular dysfunction or early atherosclerosis long before they manifest as acute clinical conditions [216]. For instance, leveraging ML on ECG data can uncover hidden phenotypes associated with CV risk, identifying individuals at risk before the onset of overt disease [205]. The application of carotid intima-media thickness (CIMT) alongside AI can enhance CV risk prediction, as it provides insights into vascular health that traditional assessments may overlook [219]. AI models that include parameters like CIMT and endothelial function can significantly improve risk stratification for patients with stable angina, helping to identify those who may benefit from more aggressive preventive strategies [215]. Additionally, innovative hybrid models that incorporate data from multiple sources, such as radiological assessments combined with clinical variables, have demonstrated the ability to predict CV risk effectively [217]. By identifying subclinical disease early, clinicians can implement targeted interventions that significantly improve long-term outcomes, emphasizing the shift towards prevention in CV care [216].

### 6.3. Population Risk Stratification and Individualized Prevention

Predictive analytics also plays a critical role in population risk stratification, allowing healthcare professionals to categorize patients based on their risk profiles and tailor preventive interventions accordingly. Traditional risk calculators often generalize risk at a population level, leading to one-size-fits-all strategies that may not adequately address the unique needs of individual patients [214]. AI-driven analytics facilitates more granular stratification by incorporating a wide array of data points, including demographic, lifestyle, and clinical characteristics, thereby enabling more individualized prevention strategies [216,220]. For example, predictive models utilizing ML have been effective in stratifying populations by identifying high-risk subgroups that might benefit from specific interventions, such as lifestyle modifications or pharmacotherapy [220]. Moreover, the integration of wearable technology into risk stratification has the potential to revolutionize preventative care. Continuous monitoring devices can collect real-time data on heart rate, rhythm, and other biomarkers, which can feed into AI models to continually refine risk assessments and inform personalized strategies for prevention and treatment [221]. For instance, patients identified by AI as having increased CV risk could be monitored with wearable devices that detect arrhythmias or other CV anomalies, allowing for timely intervention [208].

Predictive analytics for CV risk represents a paradigm shift in how CV health is assessed and managed. The integration of AI-driven models enhances traditional risk calculators, enabling early detection of subclinical disease, improving population risk stratification, and allowing for individualized prevention strategies. As technology evolves, the future of CV care will likely focus on personalized patient management, leveraging the power of predictive analytics to optimize outcomes and reduce the burden of CV diseases globally.

## 7. AI in Hypertension

Hypertension affects more than one billion individuals worldwide and remains the leading modifiable risk factor for CV morbidity and mortality, contributing substantially to coronary artery disease, HF, stroke, and chronic kidney disease. Despite advances in pharmacotherapy and prevention, global BP control rates remain suboptimal. Conventional hypertension care relies on intermittent clinic-based BP measurements and population-averaged risk models, which are limited by observer bias, measurement variability, and an inability to capture the complex, dynamic regulation of BP over time. AI offers new opportunities to improve BP measurement, risk prediction, and individualized management by integrating large-scale, multimodal data sources [222].

AI systems excel at identifying latent patterns within high-dimensional datasets, including electronic health records (EHRs), physiological waveforms, wearable sensor data, and sociodemographic variables [223]. In hypertension, these capabilities enable earlier detection, more precise risk stratification, and personalized therapeutic strategies that extend beyond traditional statistical approaches.

### 7.1. Improving BP Measurement

Accurate BP measurement is foundational to hypertension diagnosis and management, yet traditional cuff-based methods are prone to white coat effects, observer bias, and limited reproducibility. AI-enhanced signal processing has facilitated the use of physiological waveforms, most notably photoplethysmography (PPG), to estimate BP continuously and non-invasively. ML models trained on PPG-derived features have demonstrated clinically relevant accuracy in distinguishing hypertensive from normotensive individuals and estimating systolic and diastolic BP, supporting the development of cuffless and wearable BP technologies.

Automated BP measurement devices (BPMDs) represent another important advance. Automated office BP monitoring (AOBP), which acquires multiple unattended measurements, reduces observer bias and anxiety-related BP elevation, producing values that more closely reflect ambulatory BP [224,225,226,227]. Numerous BPMDs have been validated against international standards, including AAMI and ESH protocols, and have demonstrated acceptable performance across diverse clinical settings [228,229,230,231]. However, accuracy limitations persist in certain populations, such as patients with arrhythmias or advanced chronic kidney disease, emphasizing the need for population-specific validation and careful device selection [232,233,234,235,236,237].

AI models have shown strong performance in predicting incident hypertension using demographic, clinical, and lifestyle variables. AUROC values have been reported to range from approximately 0.77 to 1.00 across ML models, including support vector machines, random forests, and gradient boosting approaches [238]. Large population-based studies, particularly in East Asian cohorts, demonstrated that ML models outperform traditional regression-based risk scores in predicting 5-year hypertension incidence, with age, baseline BP, BMI, and metabolic parameters emerging as key predictors [238,239]. Beyond conventional risk factors, DL techniques can extract novel digital biomarkers from routine diagnostic data. A DL-derived biomarker from standard 12-lead ECG recordings was shown to detect prevalent hypertension and stratify future CV risk independent of overt ECG abnormalities [240]. These findings illustrate how AI can uncover subclinical disease signatures embedded in widely available clinical tests, potentially enabling earlier intervention.

### 7.2. Clinical Management and Treatment Optimization

AI applications increasingly extend into hypertension management, particularly through remote BP monitoring and treatment optimization platforms. ML-driven systems can integrate longitudinal BP data, medication history, and patient characteristics to support individualized therapy adjustment and reduce therapeutic inertia [241]. These approaches mirror developments in other chronic diseases, where predictive models guide treatment selection and titration. Medication adherence remains a major determinant of BP control and a critical target for AI intervention. ML algorithms can identify patients at high risk for non-adherence based on behavioral, clinical, and socioeconomic factors [242,243]. AI-enabled mobile applications and conversational agents have demonstrated modest but consistent improvements in adherence by delivering personalized reminders, education, and motivational feedback [243,244,245]. At the health-system level, these tools enable proactive identification of adherence barriers and targeted deployment of support resources. Wearable technologies integrated with AI have shifted BP assessment from episodic measurement toward continuous monitoring. Cuffless BP devices based on PPG and related sensors, increasingly embedded in consumer wearables, allow frequent, real-world BP estimation when appropriately calibrated [246,247,248,249,250]. Continuous data streams analyzed by ML models enable assessment of BP variability, circadian patterns, and early dysregulation that may be missed by clinic-based measurements. Wearables also enhance patient engagement by providing real-time feedback and facilitating integration with telehealth platforms [251,252,253,254]. Wearable-enabled monitoring can improve adherence and patient involvement in hypertension management. At the population level, aggregated wearable data support risk stratification, epidemiologic surveillance, and targeted interventions, particularly in remote or underserved communities [250,255]. Nevertheless, concerns remain regarding data accuracy, device calibration, validation against reference standards, and long-term usability [256,257,258,259,260,261]. Standardized validation frameworks and clear clinical integration pathways are essential to ensure that wearable BP technologies augment, rather than complicate, hypertension care. AI also supports population-level hypertension management through geospatial and behavioral analytics. Geographic information systems can identify regional disparities in hypertension prevalence, treatment access, and outcomes, enabling targeted public health interventions [262,263,264,265]. Behavioral analytics derived from EHRs, mobile applications, and wearables allow identification of adherence barriers and tailoring of interventions to individual behavioral patterns [266,267,268,269,270]. Lifestyle modification remains central to hypertension prevention. AI-enhanced digital platforms support dietary change, physical activity, and weight management by delivering personalized recommendations and monitoring progress [271,272,273,274,275,276]. When integrated with community-based programs, these tools may amplify the reach and sustainability of prevention strategies.

Despite its promise, AI implementation in hypertension care faces significant challenges, including data quality limitations, algorithmic bias, lack of external validation, limited interpretability, and regulatory uncertainty [222]. Failures of some large-scale initiatives to generalize beyond controlled environments highlight the importance of rigorous real-world evaluation. Consensus frameworks emphasize improving BP measurement fidelity, ensuring representative datasets, adopting human-in-the-loop designs, and conducting prospective clinical trials as prerequisites for scalable deployment. Thus, AI offers substantial opportunities to improve hypertension care by enhancing BP measurement, enabling early detection, personalizing treatment, and supporting population-level prevention. However, current evidence supports its role as a clinician-augmenting technology rather than a replacement for clinical judgment. Thoughtful integration, rigorous validation, and ethical governance will be essential to realize the full potential of AI in reducing the global burden of hypertension and its CV consequences.

## 8. AI in HF Management

AI is revolutionizing the management of HF through various strategies, including automated phenotyping, prognostication of readmission risk, optimization of device therapy, and facilitating remote monitoring with sensors and AI-driven alerts.

### 8.1. Automated Phenotyping: HFrEF vs. HFpEF

Automated phenotyping plays a crucial role in distinguishing between various forms of HF, particularly HF with reduced ejection fraction (HFrEF) and HF with preserved ejection fraction (HFpEF). Accurate phenotyping allows personalized treatment approaches tailored to the underlying mechanisms of each type of HF [277]. Traditional diagnostic methods often struggle to differentiate between HFrEF and HFpEF effectively, leading to misclassification and suboptimal management [278].

AI-driven models utilize ML algorithms to analyze vast amounts of clinical and biometric data to identify distinct phenotypic profiles indicative of HFrEF or HFpEF. For example, by employing echocardiographic data, laboratory values, and patient demographics, AI systems can generate predictive models that accurately classify patients’ HF types [279]. Such classification not only improves diagnostic precision but also enhances treatment strategies. For instance, patients with HFrEF may benefit significantly from specific HF medications like angiotensin-converting enzyme inhibitors (ACEis); conversely, patients categorized as HFpEF often require different management strategies focusing on comorbid conditions such as hypertension and diabetes [280].

### 8.2. Prognostication and Readmission Risk Prediction

AI models play a pivotal role in prognostication and predicting the risk of readmission among HF patients. Readmissions pose a significant burden on healthcare systems and often negatively impact patient outcomes. Data-driven AI systems can analyze numerous risk factors, including medication adherence, vital signs, and historical hospitalization records to identify patients at high risk for readmission [281].

Recent studies employing AI algorithms have successfully developed predictive models that can stratify patients based on their likelihood of experiencing adverse events such as hospital readmission or mortality. For instance, the PARTNERS-HF study utilized ML to discern aspects influencing readmissions effectively, helping clinicians tailor follow-up and management plans to the patients most at risk [282]. Incorporating social determinants of health further enhances these models, allowing for a comprehensive assessment that goes beyond clinical data, thus improving prognostic accuracy [283].

### 8.3. Optimization of Device Therapy: ICDs and CRT

The optimization of device therapy, specifically implantable cardioverter-defibrillators (ICDs) and cardiac resynchronization therapy (CRT), is being significantly enhanced through AI applications. ICDs are crucial for preventing sudden cardiac death in patients with specific risk factors, such as those with HFrEF and prior ventricular tachycardia episodes. Barriers to optimal ICD therapy adoption include patient selection criteria and the person’s overall prognosis as indicated by their left ventricular ejection fraction (LVEF) and New York Heart Association (NYHA) functional class [284,285].

AI systems are employed to analyze EHRs and real-time clinical data to refine patient selection for ICD and CRT implantation. Such algorithms can provide insights into who may benefit most from device therapy by examining various clinical endpoints and patient characteristics [286]. The use of AI-enhanced tools is also advocated to predict prognosis and determine the appropriateness of device therapy, thereby promoting improved patient outcomes [287]. Using predictive modeling could enhance the identification of patients who will gain substantial mortality risk reduction from ICD therapy compared to standard medical management, particularly in certain populations such as those with non-ischemic cardiomyopathy [288,289]. Further exploration into optimizing the placement of leads in CRT patients through AI-driven simulations has also demonstrated potential improvements in patient outcomes [290].

### 8.4. Remote Monitoring with Sensors and AI-Driven Alerts

Recent advancements in remote monitoring technologies have transformed the management of HF patients using AI. Wearable sensors and mobile devices allow healthcare providers to monitor patient vitals continuously, facilitating early detection of decompensation [291,292]. AI algorithms analyze real-time data and provide actionable insights, allowing for timely interventions, thereby preventing hospitalizations.

For example, sensors capable of tracking changes in vital signs, such as heart rate and respiratory patterns, can trigger alerts to both healthcare teams and patients when deviations from normal ranges occur [293]. Utilizing AI-driven alerts in conjunction with remote monitoring was shown to significantly reduce HF hospitalizations by allowing for swift corrective actions based on patient health data [294].

Integrating AI with telehealth platforms enables healthcare professionals to maintain continuous engagement with patients, reinforcing adherence to treatment regimens while providing the necessary support for self-management of their condition [295,296,297]. Remote monitoring facilitated by AI-powered systems effectively empowers patients, allowing them to take proactive steps toward managing their HF, fostering a sense of control over their health [298].

AI-driven strategies for HF management are pivotal in improving patient outcomes through enhanced automated phenotyping, effective prognostication, optimization of device therapy, and continuous remote monitoring. The integration of AI into HF care provides opportunities for personalizing treatment, improving coordination among healthcare teams, and fostering patient empowerment through ongoing engagement. As technology continues to advance, embracing these AI applications will be essential in redefining HF management and elevating the quality of care offered to patients.

## 9. CAD and Acute Coronary Syndromes

AI is transforming the landscape of CADs and acute coronary syndromes (ACSs) through innovative applications in chest pain triage, prognostication, imaging techniques, and the prediction of major adverse CV events (MACEs).

### 9.1. AI in Chest Pain Triage and Risk Stratification in the Emergency Department

The assessment and triage of patients presenting with chest pain in the emergency department (ED) is critical for the timely management of potential acute coronary syndromes. Traditional approaches to chest pain evaluation involve standardized clinical assessments and diagnostic tests, which may not be sufficiently sensitive to accurately identify patients at risk [299]. AI-driven models, however, can enhance risk stratification by rapidly analyzing vast amounts of clinical data, including patient demographics, medical history, laboratory results, and imaging studies to identify high-risk individuals more effectively.

For instance, ML algorithms trained on large datasets have shown promising results in predicting the risk of acute myocardial infarction (AMI) based on presenting symptoms and physiological parameters [300]. These models can assist clinicians in making more informed decisions regarding further diagnostic testing or immediate interventions. AI-enhanced triage systems can significantly reduce door-to-needle or door-to-balloon times by streamlining the process of identifying patients who require immediate treatment [301].

The deployment of AI in the ED can facilitate the standardization of care pathways for patients presenting with chest pain, enabling quicker and more accurate evaluations that lead to prompt triage decisions. For example, predictive algorithms developed by researchers have successfully risk-stratified patients with chest pain, identifying those who would benefit most from expedited invasive procedures such as cardiac catheterization or stenting [302]. This capability enhances patient care by ensuring that interventions are tailored to individual risk profiles.

### 9.2. Image-Based Assessment: Coronary Angiography, IVUS, and OCT

The role of imaging technologies in diagnosing and managing CAD and ACS has expanded significantly, particularly with advancements in intravascular imaging modalities like intravascular ultrasound (IVUS) and optical coherence tomography (OCT). While traditional coronary angiography remains the cornerstone for evaluating coronary anatomy and stenosis, IVUS and OCT offer critical insights into plaque characteristics and vascular morphology that are not always visible through angiography alone [300].

AI-driven image analysis tools can enhance the interpretation of angiograms and intravascular images by assisting in the identification and characterization of vulnerable plaques, which are associated with an increased risk of plaque rupture and acute coronary events [303]. For instance, combining IVUS and OCT approaches in assessing lesion morphology and composition could lead to better stratification of patients based on their risk profiles [304]. ML algorithms can process these imaging modalities, detecting subtle changes that may indicate impending complications, ultimately guiding timely intervention strategies.

One meta-analysis highlighted that patients receiving IVUS or OCT guidance during percutaneous coronary interventions (PCIs) experienced improved clinical outcomes compared to those managed with standard angiography methods alone, highlighting the importance of integrating advanced imaging techniques with AI tools to enhance procedural decision-making [305,306].

### 9.3. Prediction of Major Adverse CV Events (MACEs)

Predicting MACEs, including myocardial infarction, HF hospitalization, and coronary revascularization, is essential for effective CV disease management. AI has emerged as a powerful tool for calculating the risk of MACEs, drawing on extensive datasets that encompass clinical, laboratory, imaging, and demographic information. For example, researchers have developed predictive models utilizing ML techniques that analyze electronic health records to forecast the likelihood of future CV events among patients with CAD [307,308].

Integrating imaging-based assessments with predictive analytics can significantly enhance MACE prediction capabilities. In patients undergoing PCI, the incorporation of IVUS and OCT findings into AI models has improved the prognostic capacity for long-term adverse events [309]. These imaging modalities provide critical details such as plaque burden, integrity, and characteristics that traditional angiography may not capture, allowing for more precise risk stratification.

Utilizing AI models to analyze follow-up data from patients treated for ACS can improve prediction accuracy for MACEs significantly. These findings underscore the potential for AI algorithms to inform clinical decision-making and optimize post-treatment care plans based on anticipated risks [306,310]. As a result, AI’s role in risk prediction can lead to more personalized healthcare where preventative measures are tailored to individual risk profiles, ultimately reducing the burden of CV diseases.

The integration of AI in CAD and ACS demonstrates transformative potential across several domains. From enhancing chest pain triage and risk stratification in emergency settings to improving diagnostic accuracy via imaging and predicting major adverse CV events, AI technologies are redefining the standards of CV care. By incorporating advanced analytics and imaging modalities, healthcare providers can enhance patient outcomes and optimize treatment strategies tailored to individual risk profiles. Continued research and validation of these AI applications are essential to maximizing their impact within clinical practice and driving innovations in CV management.

## 10. Wearable Technologies and Remote Monitoring

The advent of wearable technologies and remote monitoring systems has significantly transformed the landscape of CV health management. This new dimension of healthcare delivery enables continuous tracking of vital signs, offers unprecedented access to real-time health data, and fosters enhanced patient engagement.

### 10.1. Smartwatches and Biosensors for Arrhythmia Detection

Smartwatches equipped with advanced biosensors represent a significant leap in the early detection and management of arrhythmias such as atrial fibrillation (AF). These devices utilize photoplethysmography (PPG) metrics to derive heart rhythm data non-invasively [311]. For instance, many smartwatches can continuously monitor heart rate and rhythm, detecting irregularities that may indicate AF, allowing for immediate reporting to the user and healthcare providers [312,313].

AI algorithms enhance these devices’ efficacy by analyzing PPG waveforms and identifying patterns linked to arrhythmias. Smartwatches can achieve diagnostic accuracy comparable to clinical standards, offering a scalable solution for arrhythmia screening. For example, smartwatch algorithms can accurately detect AF with a sensitivity of over 90% and specificity above 95% when validated against standard ECG readings [314].

The integration of real-time notifications from these wearables encourages user engagement and adherence to follow-up actions, increasing the likelihood of diagnosis and treatment initiation for arrhythmic conditions. AI-driven insights based on daily activity monitoring can prompt patients to seek medical attention when they demonstrate signs of potential arrhythmias, enhancing the cycle of care [315].

### 10.2. BP, Heart Rate Variability, Sleep, and Stress Monitoring

Wearable devices have also evolved to monitor multiple physiological parameters related to CV health, including BP, heart rate variability, sleep patterns, and stress levels. The ability to continuously track BP using cuffless devices represents a critical advancement in hypertension management. Wearable devices utilizing innovative technologies, such as PPG and ECG, can accurately estimate BP without traditional cuff methods [316,317].

Continuous BP monitoring has become an essential tool for identifying transient hypertension and managing chronic conditions. By bringing greater awareness of BP trends to patients, wearables empower them to actively participate in their health management [318]. For example, daily BP recordings from wearable devices allow users to correlate lifestyle factors (such as physical activity and sleep quality) with their BP readings, leading to more informed decisions about their health.

Heart rate variability is another key parameter measured by wearables that offers insights into the autonomic nervous system regulation and overall CV health. Monitoring heart rate variability can reveal stress levels and potential imbalances between sympathetic and parasympathetic activity, enabling users to adjust their lifestyle choices for better health outcomes. The data collated from such continuous monitoring can also assist healthcare professionals in developing individualized intervention strategies targeting stress reduction and lifestyle adjustments.

### 10.3. Digital Twins and Personalized CV Surveillance

The concept of digital twins refers to creating a virtual replica of a patient, utilizing real-time data from wearable devices and other health monitoring technologies [319]. This innovative approach allows for the simulation of various health scenarios, enabling healthcare providers to predict how patients will respond to treatments based on their unique physiological and behavioral data [319,320].

AI algorithms can analyze data patterns from wearables and medical histories to inform the virtual model, predicting the potential outcomes of different interventions. This capability is particularly valuable in CV health management, where understanding individual risks and responses to treatment can lead to truly personalized care plans [321,322]. For instance, simulations derived from digital twins can help identify optimal medication dosages or lifestyle modifications needed to mitigate hypertension risks and improve overall CV health [322].

The implementation of digital twins within telemedicine has the potential to enhance remote patient monitoring initiatives. By creating personalized digital profiles, clinicians can provide targeted, evidence-based recommendations while enabling timely adjustments to treatment based on ongoing physiological data from wearable sensors [317,323]. This level of personalized CV surveillance holds promise for improving patient adherence, promoting sustained engagement, and ultimately leading to better health outcomes.

So, the integration of wearable technologies and remote monitoring systems into CV health management presents transformative opportunities for early detection, personalized treatment, and improved patient engagement. From smartwatches facilitating arrhythmia detection to continuous monitoring of BP, heart rate variability, and lifestyle factors, these technologies enable proactive management of CV diseases. The emerging concept of digital twins further enhances personalized surveillance, paving the way for tailored interventions that respond to individual patient needs. As technology advances, the continuous refinement of wearable applications will be essential, ensuring they are accessible, reliable, and effective tools for managing CV health.

## 11. AI in Cardiac Procedures and Surgery

AI is increasingly being integrated into the field of cardiac procedures and surgery, enhancing diagnostic capabilities, optimizing treatment strategies, and improving patient outcomes. From decision support in interventional cardiology to remote patient monitoring, the role of AI continues to expand.

### 11.1. Decision Support in Interventional Cardiology

In interventional cardiology, AI-driven decision support systems play an integral role in diagnosing and treating CAD and ACS. These systems analyze vast amounts of clinical data, including patient history, test results, and procedural outcomes, to assist healthcare providers in making informed decisions during critical intervention procedures [324]. For instance, AI algorithms have been developed that can predict the likelihood of complications during PCIs, enabling cardiologists to tailor their approaches to individual patient needs, thus optimizing procedural outcomes [325].

AI systems assist in real-time decision-making by integrating data from various sources, including imaging studies, and offering recommendations on the best-practice treatment pathways. Such systems can enhance procedural efficiency and reduce adverse outcomes by providing cardiologists with actionable insights during interventions [326,327]. This use of AI not only aids in improving patient care but also fosters a more collaborative approach between clinicians and technology, ensuring that therapeutic decisions are data-driven and patient-centered.

### 11.2. Electrophysiology Mapping and Ablation Guidance

AI technologies are particularly beneficial in electrophysiology, especially for mapping and guiding ablation procedures in patients with arrhythmias. Electrophysiology mapping involves identifying areas of the heart responsible for abnormal rhythms, a process traditionally reliant on operator experience and manual interpretation of data collected during procedures. AI-enhanced mapping systems can analyze electrical signals more rapidly and accurately than human operators, improving the identification of arrhythmogenic substrates [328].

ML algorithms can process data from electrophysiological recordings, facilitating the creation of detailed maps that highlight potential targets for ablation. For example, the integration of AI in the CARTO 3 system has demonstrated improved accuracy in real-time mapping of atrial fibrillation during ablation procedures [329]. These systems not only augment procedural precision but also enhance patient safety by providing real-time feedback and alerts if abnormal patterns are detected, allowing timely intervention to prevent complications [330].

The predictive capabilities of AI can also extend to following ablation procedures, where ML models assess patient recovery paths and potential for recurrence, assisting clinicians in monitoring the long-term efficacy of treatment [331]. This comprehensive mapping and guidance provided by AI culminate in better procedural outcomes and improved long-term management strategies for patients with complex arrhythmias.

### 11.3. Robotics and AI in Cardiac Surgery

Robotic-assisted surgeries have revolutionized minimally invasive techniques in cardiac procedures, providing enhanced precision, reduced recovery times, and improved cosmesis. The da Vinci Surgical System, the most recognized robotic platform, enables surgeons to perform complex operations with enhanced dexterity and visualization [332]. The integration of AI into robotic surgery systems facilitates autonomous functions, allowing for improved surgical planning, execution, and adaptability to real-time changes in the operative environment [333].

AI applications are currently being developed to enhance robotic systems, potentially allowing for intelligent automation of repetitive tasks. For example, AI could assist by providing surgeons with enhanced data analytics for preoperative planning, analyzing anatomical structures and generating individualized surgical approaches based on a patient’s unique characteristics [334]. Robotic systems with AI capabilities can process vast amounts of intraoperative data, offering real-time feedback on patient physiology, thus allowing for dynamic surgical adjustments as needed [335,336].

The combination of robotic systems and AI applications can also facilitate training and skill assessment for new surgeons, utilizing advanced visualization and performance feedback mechanisms to enhance learning outcomes [337]. The virtuous cycle of iterative learning and performance enhancement harnessed via AI and robotics ultimately elevates patient care standards in cardiac surgery.

### 11.4. AI for Perioperative Risk Prediction

AI’s application in perioperative risk prediction represents another crucial avenue of development in cardiac procedures. Assessing the risk associated with surgical interventions is pivotal for optimizing patient outcomes and minimizing complications. Traditional risk scoring systems, while useful, often lack the granularity and predictive power afforded by AI-driven models [338].

AI algorithms can analyze preoperative data, including medical history, comorbidities, and laboratory results, to predict patient outcomes with remarkable accuracy. For instance, models that incorporate ML techniques can evaluate risk-adjusted outcomes and provide stratifications that inform surgical candidacy based on individual profiles [339,340]. These AI-based predictive models can outperform conventional models in identifying patients at heightened risk for postoperative complications, including prolonged hospitalization or adverse CV events [325,341]. The integration of AI in preoperative settings can provide surgeons with actionable insights, enabling them to explore alternative strategies or optimize interventions tailored to individual patients’ risk factors. Moreover, enhancing risk prediction accuracy contributes directly to informed consent processes, ensuring that patients are adequately educated about the potential risks and benefits of their surgical procedures [342,343].

AI is poised to significantly impact cardiac procedures and surgery through enhanced decision support, advanced electrophysiology mapping, robotic assistance, and improved perioperative risk prediction. These innovations promise to refine clinical practice, optimizing patient outcomes while reducing complications. As AI technologies continue to evolve, ongoing research and development are essential to ensure effective integration into clinical workflows, maximizing their benefits for cardiac patients and healthcare systems alike.

## 12. Pharmacotherapy and Drug Discovery

The integration of AI in pharmacotherapy and drug discovery for CV diseases has opened new avenues for improving treatment outcomes and enhancing patient adherence.

### 12.1. AI in Drug Repurposing for CV Diseases

Drug repurposing, or repositioning existing medications for new therapeutic uses, is a cost-effective strategy in drug discovery that can accelerate the availability of treatments for CV diseases. AI plays a pivotal role in this process by employing ML algorithms to analyze large biological datasets, drug interactions, and patient response profiles. For instance, AI algorithms can identify potential candidates from existing drug libraries by correlating molecular data with disease etiology, thereby facilitating the discovery of new uses for well-known medications [344].

AI models can assess molecular structures and pathways associated with CV diseases, revealing unexpected effects of drugs originally developed for other conditions. For example, antihypertensive agents have been repurposed for managing HF and drugs initially designed for diabetes treatment can be used in patients with CAD [345]. This propensity for AI to rapidly identify promising repurposing options streamlines the research process and significantly reduces the time and costs associated with traditional drug discovery pathways.

AI can help prioritize drug repurposing candidates by evaluating their pharmacokinetics, existing safety profiles, and historical efficacy data. Algorithms that assess real-world evidence and clinical trial data can provide insights into the likelihood of success for certain drugs, enabling researchers and clinicians to make informed decisions regarding potential new treatments for CV diseases [346]. The integration of AI into repurposing efforts guarantees a more streamlined and efficient path to discovering effective treatments.

### 12.2. Predicting Drug Response and Adverse Events

AI’s capacity to predict individual patient responses to medications is especially critical in the context of pharmacotherapy for CV diseases, where patient variability can significantly affect treatment efficacy [347]. ML algorithms can analyze diverse datasets, including genetic information, demographic factors, and concurrent health conditions, to develop predictive models of how patients will respond to specific medications [348].

Using AI for adverse event prediction is equally significant. Understanding which patients are at risk for drug-related side effects, such as reactions to antiplatelet therapy or anticoagulant drugs, can help healthcare providers tailor treatment plans accordingly. For example, AI-driven analysis of patient records can identify historical trends related to the adverse events associated with specific medications, allowing clinicians to engage in proactive management strategies [349]. Predictive AI models can accurately forecast adverse drug events, assisting in clinical decision-making and encouraging patient-centered care [350].

AI-driven analytics can also facilitate the identification of drug-drug interactions and patient-specific risk factors. ML models can analyze patient characteristics alongside pharmacological data to predict interactions that may exacerbate conditions or lead to adverse outcomes, thereby augmenting patient safety [351]. Such predictive capabilities underscore the potential for AI to enhance pharmacovigilance, which is essential for promoting safe medication practices in CV care.

### 12.3. AI-Based Digital Therapeutics and Medication Adherence Monitoring

AI technologies have led to the development of digital therapeutics aimed at promoting medication adherence among patients with CV diseases. These interventions utilize mobile applications, web platforms, and wearable devices to monitor patients’ medication-taking behaviors in real-time and provide customized reminders, educational content, and motivational support [352].

AI-based interventions can significantly enhance adherence rates. For instance, integrating a smartphone application that utilizes AI algorithms to analyze data related to medication adherence has been reported to improve adherence rates among patients prescribed antihypertensive medications [353]. By sending personalized alerts according to individual medication regimens, these applications can reduce forgetfulness, which is a common barrier to medication adherence [352].

Digital therapeutics can implement behavioral nudges based on AI analytics, which adapt to patients’ unique patterns and preferences. These personalized strategies align with the principles of behavioral economics, fundamentally altering how patients perceive their medication regimens and fostering consistent adherence behaviors [354]. By aiding patients in tracking their health metrics and engaging them in goal-setting, these digital therapeutics empower individuals to take control of their CV health, promoting preventive care and reducing the risk of acute events due to non-adherence.

AI-driven strategies in pharmacotherapy and drug discovery for CV diseases promise to transform the management of treatment regimens and improve patient outcomes. AI’s potential for drug repurposing, predicting drug responses and adverse events, and supporting digital therapeutics for medication adherence monitoring clearly illustrates its value across the CV spectrum. As technology continues to evolve, the integration of AI in pharmacotherapy will likely promote personalized medicine approaches, enhance adherence rates, and foster better management of CV diseases.

## 13. Genomics, Multi-Omics, and Precision Cardiology

The integration of genomics, proteomics, and metabolomics with AI marks a significant advancement in precision cardiology. This convergence enables a deeper understanding of CV diseases and provides innovative pathways for personalized treatment strategies.

### 13.1. Integration of Genomics, Proteomics, Metabolomics with AI

The incorporation of omics technologies (including genomics, proteomics, and metabolomics) creates a comprehensive dataset that reflects the intricate pathophysiology of CV diseases. AI techniques facilitate the analysis of these extensive datasets, enabling the identification of patterns challenging to discern through conventional statistical methods. For instance, genomic sequencing can uncover genetic variants associated with elevated CV risk, while proteomic and metabolomic studies can highlight changes in protein expression and metabolite concentrations indicative of disease progression [327,355].

AI-driven algorithms, particularly those utilizing ML and DL methodologies, excel at processing high-dimensional data across various omic layers to extract meaningful insights. Models based on genomic data can predict individual patient responses to treatments based on their genetic backgrounds, thereby assisting in tailoring therapies [356]. Additionally, integrating proteomic data with clinical information through AI analysis can enhance classification accuracy for HF phenotypes and other CV conditions [252]. This multi-omic framework improves diagnostic pathways by providing a more holistic overview of biological processes in CV patients.

### 13.2. Identification of Novel Biomarkers and Therapeutic Targets

AI’s analytical capabilities play a crucial role in discovering novel biomarkers and therapeutic targets for CV diseases. Biomarkers serve essential functions in diagnostics, prognostics, and assessing therapeutic responses. AI can expedite biomarker discovery by scrutinizing extensive datasets from clinical trials, patient registries, and laboratory findings [357]. For example, ML algorithms have been employed to identify biomarkers related to HF, including specific microRNAs that correlate with disease severity and therapeutic response [358].

Moreover, AI facilitates the identification of potential therapeutic targets by examining gene expression patterns and proteomic data to uncover molecules suitable for therapeutic modulation. A notable example includes the identification of pyruvate kinase M2 as a potential regulator in atherosclerosis, with AI models indicating how targeting this enzyme could create new therapeutic opportunities [359]. By streamlining the discovery and validation of such targets, AI-driven approaches significantly enhance the toolkit available for managing CV diseases.

### 13.3. Personalized Care Guided by AI-Enabled Omics Analysis

AI’s integration into precision cardiology ultimately manifests in its ability to tailor patient care through AI-enabled omics analyses. This personalized approach aligns treatment protocols with the unique genetic, proteomic, and metabolomic profiles of individual patients, allowing for targeted therapies that optimize clinical outcomes [360]. For instance, using genomic data analysis, clinicians can select medications that are predicted to be more effective based on the patient’s genetic profile, thereby reducing the likelihood of adverse reactions and improving therapeutic efficacy [361].

AI can continuously refine treatment strategies by utilizing real-time patient data. Digital health technologies, including wearable devices that monitor physiological parameters, can supply data to AI algorithms that continuously update patient profiles [362]. This dynamic feedback loop offers clinicians the flexibility to modify treatment plans based on ongoing assessments of CV health and therapy responses, which can enhance adherence and patient engagement [363].

AI-driven platforms that visualize multi-omics data empower healthcare providers to interpret complex biological interactions intuitively, allowing for tailored interventions based on a comprehensive understanding of each patient’s health condition [364]. As healthcare shifts toward personalized medicine, the integration of AI with omics data will be critical for enhancing precision cardiology and effectively managing CV diseases.

The synergy between AI and multi-omics delineates a transformative pathway in precision cardiology that improves diagnostic accuracy, personalizes treatment strategies, and fosters the identification of novel biomarkers and therapeutic targets. As the field evolves, the integration of AI with genomic, proteomic, and metabolomic data will further enhance our understanding of CV diseases while promoting individualized healthcare interventions. Ongoing research and development in these technologies will be paramount for optimizing patient outcomes and shaping the future of CV medicine.

## 14. Ethical, Legal, and Social Implications of AI in Cardiology

The integration of AI into cardiology presents substantial ethical, legal, and social implications. As AI technologies become more embedded in clinical settings, it is essential to understand the challenges they pose, including algorithmic bias, data privacy and cybersecurity concerns, and the need for robust regulatory frameworks and clinical validation requirements.

### 14.1. Algorithmic Bias and Health Disparities

One of the significant concerns surrounding the deployment of AI in healthcare is algorithmic bias, which can perpetuate or even exacerbate existing health disparities. AI algorithms are trained on datasets that may reflect historical biases in patient care, potentially leading to unequal healthcare outcomes across different demographic groups [365,366]. Concerns related to biased algorithms are particularly relevant in cardiology, where CV diseases disproportionately affect specific populations, including racial and ethnic minorities, women, and older adults [367].

ML models can exhibit varying predictive accuracy based on demographic variables, such as gender and ethnicity, potentially leading to misdiagnosis or inappropriate treatment recommendations for certain groups [368]. For example, disparities in data representation can lead to algorithms that are less effective at identifying risk factors and making accurate predictions for underrepresented populations. This raises ethical questions about fairness in treatment and the provision of care [369].

Addressing algorithmic bias requires the integration of diverse and representative datasets when training AI models, ensuring that these tools reflect the heterogeneity of patient populations. Ongoing efforts to audit algorithms for bias and implement fairness-enhancing interventions will be crucial in maintaining equitable healthcare practices in cardiology [370]. Involving multidisciplinary teams of clinicians, ethicists, and data scientists in the development and evaluation phases can mitigate bias and enhance accountability [371].

### 14.2. Data Privacy, Cybersecurity, and Patient Trust

The proliferation of AI technologies in cardiology raises significant concerns regarding data privacy and cybersecurity. As AI systems process vast amounts of sensitive patient information, including EHRs, there is an increasing risk of data breaches and unauthorized access [372]. Protecting patient data is essential for maintaining confidentiality and trust in healthcare systems, particularly as patients become more aware of the potential vulnerabilities associated with digital health technologies.

To navigate these complexities, healthcare institutions must prioritize robust cybersecurity measures that safeguard patient data against breaches. Implementing encryption, access controls, and regular security assessments will be vital for protecting sensitive information [373]. Transparency regarding how patient data is utilized in training AI models is crucial for building trust between patients and healthcare providers. Patients should be informed about what data is collected, how it is processed, and how it influences clinical decisions, fostering an environment of openness and accountability [374].

Establishing comprehensive policies that govern data sharing and utilization in AI research is necessary to navigate ethical concerns associated with patient consent and data ownership [375]. Regulatory frameworks should be established to guide the ethical use of AI within healthcare, ensuring that patient rights are upheld while promoting innovation in patient care.

### 14.3. Regulatory Frameworks and Clinical Validation Requirements

As AI applications proliferate in cardiology, establishing robust regulatory frameworks and clinical validation requirements is paramount. Regulatory bodies need to formulate guidelines regarding the development, testing, and deployment of AI technologies in clinical settings, similar to the frameworks established for medical devices and pharmaceuticals [376]. The European Union’s AI Act, for example, emphasizes transparency and accountability while addressing risks associated with AI deployment. Clinical validation of AI algorithms is essential to establish their efficacy and safety in real-world healthcare scenarios. This process often requires rigorous testing against standard clinical practices to assess performance across diverse patient populations [377,378,379]. Engaging multidisciplinary and external oversight, including diverse stakeholders in the healthcare ecosystem, can provide critical insights during the validation process, ensuring that algorithms are applicable and relevant to clinical practice [380]. As AI-enabled technologies become integrated into everyday clinical workflows, continuous monitoring of their performance is critical. Establishing feedback loops for post-deployment assessments to track AI effectiveness and patient outcomes will allow healthcare systems to adapt quickly based on real-world evidence [372,381]. Such an iterative approach will be essential to maximize the benefits of AI in cardiology while ensuring that patient safety and ethical standards remain at the forefront.

The ethical, legal, and social implications of AI in cardiology necessitate careful consideration as these technologies become more entrenched in the clinical landscape. Addressing algorithmic bias, ensuring data privacy and cybersecurity, and establishing clear regulatory frameworks and clinical validation requirements are paramount to fostering equitable and safe AI-enabled CV care. By prioritizing these aspects, healthcare providers can harness the transformative potential of AI while ensuring that the advancements genuinely enhance patient care and outcomes.

## 15. Challenges and Barriers to Clinical Adoption

The use of AI in cardiology has significantly enhanced diagnostic capabilities, treatment outcomes, and patient management. However, challenges and barriers to the clinical adoption of AI technologies remain.

### 15.1. Data Quality, Reproducibility, and Generalizability

The effectiveness of AI in clinical applications largely depends on the quality of the data used to train ML algorithms. High-quality data is essential for developing robust and reliable AI models capable of yielding accurate predictions and diagnoses. Many datasets, however, contain missing values, errors, or inconsistencies that can introduce biases and affect the model’s performance [381]. This issue is particularly pronounced in cardiology, which often relies on varied and heterogeneous data sources, including EHRs, imaging data, and genomic profiles. Inconsistent data formats and terminologies across institutions exacerbate these challenges, leading to concerns about the reproducibility of AI findings and generalizability to diverse patient populations.

AI algorithms trained on data from specific cohorts may not perform equivalently when applied to broader populations due to variations in demographics and clinical presentations. For instance, an AI model developed using an urban population may not accurately predict CV events in rural settings, emphasizing the need for diversity in training datasets. Addressing these issues involves improving data collection methodologies and implementing standardized protocols for data-sharing across institutions [381].

### 15.2. Integration into Clinical Workflow and EHR Systems

Integrating AI technologies into existing clinical workflows and EHR systems presents another significant barrier. AI applications must be seamlessly incorporated into the daily practices of healthcare professionals to enhance rather than disrupt their work [382]. When AI tools are introduced without consideration for clinical workflow, there is a risk of clinician resistance, confusion, and decreased efficiency.

For successful integration, AI tools should complement clinical decision-making processes, providing clinicians with actionable insights without overwhelming them with complex outputs. User-friendly interfaces that facilitate easy interaction with AI algorithms can promote the adoption and utilization of these technologies in practice [383]. Training healthcare professionals to use AI tools effectively alongside their clinical expertise is essential for optimizing AI’s benefits in cardiology [367,384].

Compliance with regulations surrounding EHR interoperability and data security must be addressed to ensure that AI tools can access necessary patient data while adhering to privacy standards. Collaborative initiatives engaging stakeholders, including healthcare professionals, software developers, and regulatory bodies, can facilitate the development of integrated solutions that promote effective AI adoption [366,385].

### 15.3. Physician Acceptance and AI Literacy in Cardiology Training

The acceptance of AI technologies among healthcare professionals is crucial for successful clinical adoption. Many clinicians may be apprehensive about AI’s role in decision-making, fearing that it could undermine their expertise or result in a dependence on technology [198]. Bridging this gap requires comprehensive educational initiatives aimed at enhancing AI literacy among healthcare providers, demonstrating how these technologies can support rather than supplant clinical judgment.

Training programs in cardiology should include AI fundamentals, enabling practitioners to understand the mechanisms, capabilities, and limitations of AI systems [368]. Clinicians engaged in AI-related research or development are more likely to embrace these technologies, fostering a culture of innovation and openness to change within the medical community [386]. Involving clinicians in the development process of AI applications ensures that these tools address practical clinical needs and incorporate expert feedback, encouraging acceptance among potential users [308,387].

While the potential for AI in cardiology is substantial, several challenges and barriers must be addressed for its successful clinical adoption. Ensuring data quality, addressing integration issues within clinical workflows and EHR systems, and fostering physician acceptance through education and training are critical areas needing ongoing attention. By navigating these hurdles collaboratively, the healthcare community can leverage AI to enhance CV care, improve patient outcomes, and usher in a new era of precision medicine.

## 16. Future Perspectives: Toward an AI-Augmented Cardiologist

The future of cardiology is characterized by the increasing integration of AI in clinical practice, leading to the emergence of AI-augmented cardiology. This transformation hinges on the collaboration between human expertise and AI technologies, fostering new opportunities for improved patient care.

### 16.1. The Role of Hybrid Intelligence (Human + AI Collaboration)

Hybrid intelligence, defined as the collaborative synergy between human clinicians and AI systems, is essential for optimizing decision-making processes in cardiology. Clinicians possess invaluable clinical expertise, intuition, and the ability to consider the nuances of individual patient circumstances. AI can complement these strengths by providing rapid data analysis, identifying patterns within complex datasets, and supporting diagnostic accuracy [388].

For instance, AI-powered tools can analyze extensive datasets from EHRs, imaging studies, and genetic information to deliver insights that enhance clinical judgment. This partnership allows cardiologists to focus on their strengths, such as patient interactions and assessments, while AI diligently parses data and identifies potential issues [389]. Clinicians utilizing AI decision support tools are more likely to arrive at timely, accurate diagnoses, ultimately improving patient outcomes [390].

Integrating AI in the clinician’s workflow can help mitigate cognitive overload in decision-making processes, making the practice of cardiology more efficient and data-driven. Building trust in AI-assisted systems is paramount for their acceptance; ongoing education and training in utilizing these tools will be crucial for fostering collaboration [391].

### 16.2. Prospects of Fully Autonomous Systems vs. Decision Support

While the current focus is on hybrid intelligence, discussions surrounding fully autonomous AI systems in cardiology are gaining traction. Fully autonomous AI systems hold the potential to revolutionize CV care through their ability to process complex information rapidly and make independent decisions; however, such systems have yet to be widely implemented in clinical settings [392].

AI platforms, once equipped for autonomous operation, may analyze patient data and diagnostic results, providing treatment recommendations based on algorithms that continually learn from new data. However, ethical considerations, such as accountability and transparency, remain critical issues that need to be addressed before physicians could reliably depend on these systems entirely [393].

On the other hand, decision support tools, which utilize AI to assist in clinical scenarios, will likely prevail in the near term. By providing clinicians with decision-enhancing insights rather than replacing their expertise, decision support systems will continue to thrive in clinical practice. This balanced approach enables AI to act as a partner in decision-making rather than a fully independent agent, ensuring that human clinical judgment remains essential in patient care [375].

### 16.3. Long-Term Vision: Reshaping Research, Education, and Clinical Care

The long-term vision for AI in cardiology envisions an environment where research, education, and clinical practice are seamlessly integrated. As the utilization of AI continues to grow, the landscape of CV research will also evolve, emphasizing the need for interdisciplinary collaboration among cardiologists, data scientists, and bioinformatics specialists [390]. The ability to combine diverse types of data, from large-scale genetic studies to real-time monitoring data, will facilitate innovative discoveries and enhance the understanding of CV diseases.

Education and training programs must also adapt to prepare future cardiologists for a landscape valuing hybrid intelligence and integration of AI tools. Curricula need to encompass foundational knowledge of AI principles, data analytics, and ethical considerations surrounding AI applications in clinical settings [394]. By equipping cardiologists with the necessary skills to navigate AI-driven environments, the field can ensure that these technologies are used responsibly and effectively [395].

In clinical care, the shift towards AI-augmented cardiology offers the promise of enhancing patient outcomes through precision medicine approaches tailored to individual profiles. AI-enabled predictive analytics can inform treatment decisions, enhance risk stratification, and support patient engagement in self-management [396]. Ultimately, fostering a culture of innovation and collaboration will be essential for realizing the full potential of AI in cardiology, thereby improving the quality of care delivered to patients [397,398,399,400].

## 17. Conclusions

AI is undeniably reshaping CV medicine, offering unprecedented opportunities to improve diagnosis, risk prediction, and patient management. Importantly, AI should not be framed as a replacement for clinical judgment, but rather as a complementary partner that augments human expertise through data-driven insights and precision analytics. Yet, the clinical impact of AI in cardiology remains heterogeneous, varying substantially across applications and stages of maturity. A realistic appraisal of AI’s role therefore requires stratification by clinical readiness, alongside a candid acknowledgment of failed translations, unmet needs, and implementation challenges.

Ranking AI applications by clinical readiness provides a pragmatic lens through which to distinguish technologies that meaningfully improve care today from those that remain investigational. High-readiness applications are already embedded in routine or near-routine clinical practice, particularly in electrocardiography and cardiac imaging. These tools have progressed beyond proof-of-concept and are now integrated into commercial ECG systems and consumer wearables, illustrating a rare alignment of technical performance, workflow integration, and clinical utility. Similarly, AI-driven automation in echocardiography, particularly for left ventricular ejection fraction quantification, has achieved strong concordance with expert readers. Beyond accuracy, these systems improve reproducibility and significantly reduce interpretation time, addressing longstanding sources of interobserver variability in imaging-based decision-making. These examples illustrate that AI adoption accelerates when algorithms solve clearly defined clinical problems, operate within established workflows, and deliver immediate, measurable efficiency gains. However, widespread clinical uptake has been constrained by limited prospective validation, unclear thresholds for actionability, and poor integration into clinician decision pathways. These challenges underscore a recurring theme in AI deployment: predictive accuracy alone is insufficient without a clear linkage to clinical decisions and outcomes. Low-readiness applications remain largely confined to research settings. Concepts such as digital twins, reinforcement learning-guided therapy optimization, and multi-omics driven decision support represent compelling visions for the future of personalized CV care.

Realizing the full clinical promise of AI in cardiology requires a deliberate and structured translational roadmap. One of the most persistent barriers remains reimbursement. AI tools are rarely reimbursed as standalone interventions, limiting institutional incentives for adoption and sustained maintenance. Progress will depend on alignment with value-based care models and incorporation into existing CPT or DRG frameworks, ensuring that AI-enabled efficiencies and outcome improvements are economically recognized. Equally critical is clinician education and AI literacy. Rather than emphasizing coding proficiency, training programs should focus on the interpretation, limitations, bias recognition, and ethical implications of AI outputs. Preparing cardiologists to critically engage with AI tools will be essential for the safe and effective integration into practice.

Looking ahead, several priority directions are poised to define the next decade of AI in cardiology. First, prospective, randomized trials evaluating AI as an intervention (rather than merely a predictor) are essential to demonstrate improvements in clinical outcomes and cost-effectiveness. Second, explainable and trustworthy AI must evolve beyond post hoc interpretability toward models that are transparent by design, enabling clinicians to understand and interrogate algorithmic reasoning. Third, equity-aware AI development, grounded in diverse and global datasets, is critical to avoid widening CV health disparities. Fourth, continuous learning systems capable of safe post-deployment updating will better reflect real-world clinical dynamics. Finally, the integration of multimodal and longitudinal data (spanning imaging, genomics, wearables, and social determinants of health) will be central to achieving truly personalized CV care.

Ultimately, the trajectory of AI in cardiology points toward a future of integrative, data-enriched, and patient-centered care. Patients are increasingly receptive to AI technologies when they enhance diagnostic accuracy and decision-making support, yet concerns about depersonalization and loss of human contact persist. Maintaining empathy, transparency, and clinician oversight within AI-augmented care models will therefore be essential to fostering public trust. When deployed thoughtfully and evaluated rigorously, AI has the potential to transform CV medicine, advancing diagnostic precision, improving outcomes, and enabling a more equitable and efficient delivery of care. The challenge now lies not in proving what AI *can* do, but in ensuring that it is implemented responsibly, ethically, and in service of real-world CV health.

## Figures and Tables

**Table 1:** Timeline of Major Discoveries in AI in Medicine.

Year	Milestone	Key Contribution to Medicine	Significance
1950	Alan Turing publishes “*Computing Machinery and Intelligence*” [1]	Conceptual foundation of machine intelligence	Introduced the idea of machines performing intelligent reasoning (“Turing Test”)
1960s	Development of early medical expert systems	Diagnostic reasoning using “if–then” rules	Established the first use of AI for medical diagnosis, particularly infectious diseases
1970s	*Internist-I* and *CASNET* introduced [2]	Knowledge-based systems for complex diseases	Showed feasibility of computer-assisted diagnosis in internal medicine
1980s	Rise of statistical and probabilistic models (Bayesian networks) [3,4,5,6]	Early models of clinical decision support and diagnostic probability	Moved AI from rigid rule-based logic toward probabilistic reasoning
1990s	Machine learning algorithms gain traction [7,8,9]	Predictive analytics for outcomes, survival, and risk modeling	Shifted focus from knowledge-based systems to data-driven learning
Early 2000s	Widespread digitization of healthcare data and electronic heath records (EHRs) [10,11,12,13,14,15,16,17,18]	Availability of structured and unstructured data for AI training	Enabled large-scale clinical data mining and natural language processing
2010–2012	Deep learning breakthroughs (ImageNet competition) [19,20]	Convolutional neural networks (CNNs) applied to medical imaging	Sparked the modern AI revolution in radiology, pathology, and ophthalmology
2014	Generative models introduced (e.g., generative adversarial networks, GANs) [21,22]	Creation of synthetic medical data and image augmentation	Allowed realistic simulations, training datasets, and privacy-preserving modeling
2015–2017	Natural language processing (NLP) advances with deep learning [23,24,25,26,27,28,29,30,31,32,33,34,35,36,37,38]	Extraction of information from clinical notes and literature	Improved automatic chart review and clinical documentation analysis
2018	FDA clears first autonomous AI diagnostic device (IDx-DR for diabetic retinopathy) [39,40,41,42,43,44,45,46]	Automated retinal screening without clinician oversight	Marked regulatory acceptance of autonomous AI in clinical practice
2019–2020	Federated learning and privacy-preserving AI gain prominence [12,47,48]	Multi-institutional model training without data sharing	Advanced collaboration while maintaining patient data privacy
2021	Transformer-based large language models (e.g., GPT, BioBERT) adapted for medicine [49,50]	Clinical text summarization, guideline synthesis, and chatbot applications	Expanded AI’s reach to clinical reasoning and communication tasks
2022–2023	Multimodal AI integrating imaging, genomics, and EHR data	Unified models for disease prediction and phenotype discovery	Enabled comprehensive “digital twin” modeling of patients
2024–Present	Generative AI and explainable AI in clinical workflows	Synthetic patient modeling, explainable diagnostics, interactive decision support	Moving toward trustworthy, human-centered, and interpretable AI in medicine

**Table 2:** Types and Dimensions of AI in Medicine.

AI Type	Key Characteristics	Typical Algorithms	Main Clinical Applications	Advantages	Limitations	Common Data Sources	Ref.
Rule-Based (Expert) Systems	Deterministic logic using “if–then” rules and domain knowledge	Decision trees, logical inference engines, ontology-based systems	Clinical decision support (diagnostic assistance, drug interaction checking, alerts)	Transparent reasoning, interpretable outputs, easy validation	Rigid, difficult to scale, cannot learn from new data	Structured EHR data, clinical guidelines, ontologies	[71]
Machine Learning (ML)	Learns predictive patterns from structured or semi-structured data	Logistic regression, Random Forests, SVM, Gradient Boosting	Risk prediction, diagnostic classification, outcome forecasting	Improves with data, identifies nonlinear patterns	Opaque decision-making, risk of bias, needs retraining	EHRs, lab data, registries, claims databases	[72]
Deep Learning (DL)	Multi-layer neural networks that automatically learn features	CNNs, RNNs, Transformers, autoencoders	Imaging (radiology, pathology), ECG/EEG interpretation, genomics	State-of-the-art accuracy, handles complex high-dimensional data	Requires large, labeled datasets, low interpretability, high cost	Imaging (CT, MRI, ultrasound), signal data, genomics	[73]
Natural Language Processing (NLP)	Interprets and generates human language from unstructured text	Named Entity Recognition, LLMs (BERT, GPT), text mining	Clinical note analysis, report generation, EHR summarization, literature mining	Unlocks unstructured text data, improves documentation	Ambiguity of medical language	Clinical notes, reports, literature, discharge summaries	[26]
Reinforcement Learning (RL)	Learns optimal strategies by interacting with environments	Q-learning, Deep Q Networks, Policy Gradients	Personalized therapy (sepsis, insulin, ventilator settings), robotic surgery control	Adaptive decision-making, continuous optimization	“Trial-and-error” not always safe in clinical settings	Patient trajectories, monitoring data, simulated environments	[74]
Computer Vision (CV)	Interprets and analyzes visual or imaging data	CNNs, Vision Transformers, object detection, segmentation	Radiology, dermatology, ophthalmology, histopathology	High diagnostic accuracy, reproducibility	Domain shift, device dependency, annotation cost	Imaging (X-ray, CT, MRI), microscopy, endoscopy	[75]
Predictive Analytics	Forecasts future outcomes using historical data	Ensemble ML, survival analysis, time series modeling	Predicting readmission, mortality, treatment response	Enables proactive care, supports planning	Needs longitudinal data, continuous validation	Registries, EHR, wearables, claims data	[76]
Robotics and Intelligent Systems	Integrates AI with mechatronics for perception and control	Control algorithms, reinforcement learning, motion planning	Robotic surgery, rehabilitation, telepresence, logistics	Precision, consistency, fatigue-free operation	Costly, technical maintenance, complex validation	Sensor data, imaging, proprioception, video	[77]
Generative AI	Creates new, synthetic data or content similar to real data	GANs, diffusion models, LLMs	Synthetic data generation, image enhancement, virtual patient modeling, report drafting	Supports simulation, training, data augmentation	Risk of fabricated data or “hallucination” [78,79,80,81]	Real-world datasets, text corpora, imaging	[82]
Federated and Privacy-Preserving AI	Learns collaboratively without sharing raw data	Federated learning, differential privacy, encryption	Multi-institutional model training	Preserves patient privacy, supports collaboration	Communication overhead, data heterogeneity	Distributed hospital data, imaging networks	[83]
Causal and Explainable AI (XAI)	Focuses on causal inference and interpretability	SHAP, LIME, causal Bayesian networks	Treatment-effect modeling, interpretable predictions	Improves trust, regulatory compliance	Limited maturity, high methodological complexity	Any structured data with defined relationships	[84]
Hybrid AI (Symbolic + Statistical)	Combines logic-based reasoning with ML adaptability	Neuro-symbolic systems, knowledge graphs, ontology integration	Clinical reasoning, multimodal diagnostics, guideline automation	Balanced interpretability and learning capacity	Integration complexity, computational intensity	Structured + unstructured data, ontologies	[85]

**Table 3:** Applications of AI in CV Imaging.

Imaging Modality	Primary AI Applications	Specific AI Functions	Clinical Benefits and Impact	Limitations	Ref.
Echocardiography	Automated measurements and disease detection	Automated quantification of LVEF, LV volumes, LA size, and mitral valve area using ML/DL algorithms Automated 3D echocardiography improving consistency over 2D methods Disease detection via tricuspid regurgitation jet analysis for pulmonary hypertension Early recognition of subtle morphologic abnormalities suggestive of HF or valvular disease Application in pediatrics and ECMO patients for reproducible assessment	Rapid and reproducible quantification of cardiac function Reduced operator dependency and improved workflow Enhanced early diagnosis and patient risk stratification Empowerment of non-expert operators in diverse clinical settings	Dependence on image quality and acquisition technique Limited generalizability across vendors and populations Need for expert oversight to ensure clinical accuracy	[133,134,135]
Cardiac Computed Tomography (CT)	Plaque characterization and coronary anatomy analysis	ML and DL models for automated identification of calcified, non-calcified, and mixed plaques Detection of coronary stenosis and risk stratification Automated lumen segmentation and quantitative coronary plaque burden analysis Real-time lesion detection and reporting	Improved diagnostic consistency and inter-reader agreement Enhanced speed and accuracy in CAD diagnosis Facilitates precision in treatment planning and risk prediction	Radiation exposure considerations Image artifacts and motion sensitivity Need for multicenter validation and regulatory standardization	[136,137,138,139,140,141,142,143,144,145,146,147,148,149]
Cardiac Magnetic Resonance Imaging (MRI)	Tissue characterization and functional analysis	AI-based LV segmentation for automated volumetric analysis Automated assessment of myocardial strain and perfusion Interpretation of late gadolinium enhancement for infarct and fibrosis mapping Differentiation between ischemic and non-ischemic cardiomyopathies	Improved precision and reproducibility of quantitative measures Reduced analysis time and reader fatigue Enhanced ability to characterize tissue pathology and predict outcomes	Computational intensity and processing time Limited interpretability of black-box DL models Need for continued clinician oversight for clinical correlation	[150,151,152,153,154,155,156,157,158,159]
Nuclear Imaging (PET/SPECT)	Perfusion quantification and hybrid imaging integration	AI-assisted quantification of myocardial perfusion and viability Automated detection of perfusion defects and volumetric blood flow mapping Integration with CT or MRI (hybrid imaging) for comprehensive anatomical-functional analysis AI-based visualization tools enhancing fusion imaging interpretation	Increased diagnostic accuracy and reproducibility Enhanced sensitivity/specificity for CAD detection Improved guidance for revascularization and therapy decisions Supports multi-modality image integration	Dependence on quality and calibration of nuclear data High computational and infrastructural requirements Limited access to multimodal datasets for algorithm training	[160,161,162,163,164,165,166]
Cross-Modality Integration	Multimodal data fusion and clinical workflow optimization	Combining imaging data (Echo, CT, MRI, PET/SPECT) with EHRs and clinical metadata Unified AI models for patient-specific “digital twin” generation Automated report synthesis and cross-validation between modalities	Comprehensive evaluation of structure, function, and perfusion Reduced diagnostic fragmentation Personalized, patient-centered imaging interpretation	Integration complexity, interoperability challenges Data harmonization and privacy concerns Regulatory and ethical oversight required	[167,168,169]

## Data Availability

No new data were created or analyzed in this study.
